# Long‐term declines in winter body mass of tits throughout Britain and Ireland correlate with climate change

**DOI:** 10.1002/ece3.4812

**Published:** 2018-12-26

**Authors:** Euan N. Furness, Robert A. Robinson

**Affiliations:** ^1^ Clare College University of Cambridge Cambridge UK; ^2^ British Trust for Ornithology, The Nunnery Thetford Norfolk UK

**Keywords:** body mass regulation, climate change, Paridae, predation risk, supplementary feeding, trade‐off, wing length

## Abstract

The optimum body mass of passerine birds typically represents a trade‐off between starvation risk, which promotes fat gain, and predation pressure, which promotes fat loss to maintain maneuvrability. Changes in ecological factors that affect either of these variables will therefore change the optimum body masses of populations of passerine birds. This study sought to identify and quantify the effects of changing temperatures and predation pressures on the body masses and wing lengths of populations of passerine birds throughout Britain and Ireland over the last 50 years. We analyzed over 900,000 individual measurements of body mass and wing length of blue tits *Cyanistes caeruleus*, coal tits *Periparus ater*, and great tits *Parus major* collected by licenced bird ringers throughout Britain and Ireland from 1965 to 2017 and correlated these with publicly available temperature data and published, UK‐wide data on the abundance of a key predator, the sparrowhawk *Accipiter nisus*. We found highly significant, long‐term, UK‐wide decreases in winter body masses of adults and juveniles of all three species. We also found highly significant negative correlations between winter body mass and winter temperature, and between winter body mass and sparrowhawk abundance. Independent of these effects, body mass further correlated negatively with calendar year, suggesting that less well understood dynamic factors, such as supplementary feeding levels, may play a major role in determining population optimum body masses. Wing lengths of these birds also decreased, suggesting a hitherto unobserved large‐scale evolutionary adjustment of wing loading to the lower body mass. These findings provide crucial evidence of the ways in which species are adapting to climate change and other anthropogenic factors throughout Britain and Ireland. Such processes are likely to have widespread implications as the equilibria controlling evolutionary optima in species worldwide are upset by rapid, anthropogenic ecological changes.

## 1 INTRODUCTION

There is strong evidence indicating that the mean body mass of bird populations can change over periods of a few years (Gosler, 2002; Gosler, Greenwood, & Perrins, 1995; Price, Grant, Gibbs, & Boag, 1984), as well as fluctuating from day to day (Broggi, Koivula, Hohtela, & Orell, 2017; Newton, 1969). One mechanism responsible for these changes involves the deposition of more body fat in colder weather, as insulation and insurance against reduced and unpredictable food availability (Krams et al., 2010; Newton, 1969, 1998). Another may be the deposition of more fat under conditions of greater disease risk, in order to buffer against reduced foraging efficiency as a result of disease (Speakman, 2018). However, these benefits come at a cost, because increased mass leads to decreased maneuvrability and therefore increased predation risk (McNamara & Houston, 1990). Consequently, birds will maintain smaller fat reserves when predators are abundant than when predators are scare (Lilliendahl, 1997). Climate change associated with increasing winter temperatures (Jenkins, Perry, & Prior, 2008) may thus be expected to lead to a reduction in body mass in small birds, because the trade‐off between higher body mass to compensate for cold‐weather‐induced starvation and lower body mass to reduce predation risk will shift away from starvation. There is also strong evidence to suggest that both natural selection (Bosse et al., 2017) and adaptive phenotypic plasticity (Gosler et al., 1995) can cause bird phenotypes to change over periods of only a few years.

Blue tits *Cyanistes caeruleus*, great tits *Parus major*, and coal tits *Periparus ater* throughout Britain and Ireland make extensive use of garden bird‐feeders and are major prey items of sparrowhawks *Accipiter nisus* (Perrins, 1979). It is therefore reasonable to expect that all three species could be affected similarly by changes in climate, predation risk, and other factors such as supplementary feeding practices, which have been shown to affect the phenotypes of garden birds (Plummer, Bearhop, Leech, Chamberlain, & Blount, 2018). Because small bird species in temperate climates rely on temperature as a cue for regulating body mass (Newton, 1969), anthropogenic increases in the mean winter temperature are likely to lead to predictable decreases in mean winter body mass in small birds. However, these changes may be quite small and long term, and therefore not easy to observe without access to large datasets.

Sparrowhawk abundance in Britain was severely reduced by organochlorine pesticide poisoning in the 1960s (Newton, 1986); however, the population recovered rapidly after the regulation of these pesticides and has since plateaued (Robinson et al., 2016). This recovery coincided with a significant drop in body mass among great tits, which occurred in specific regions at the same time as sparrowhawk populations recovered in those regions (Gosler et al., 1995). Increases in sparrowhawk abundance across Britain might therefore also be expected to coincide with a decrease in body mass of tits.

Supplementary feeding of garden birds has increased in recent decades, with notable effects on the ecologies of some species, such as the Eurasian blackcap *Sylvia atricapilla* (Plummer, Siriwardena, Conway, Risely, & Toms, 2015). Supplementary feeding increases food availability and predictability, especially in winter, and would therefore be expected to lead small birds such as tits to downregulate their winter fat deposits, thus reducing their winter body mass.

In this study, we used the huge long‐term datasets generated by citizen science bird ringing in Britain and Ireland to analyze seasonal and long‐term changes in mean adult and juvenile body masses in three abundant tit populations (blue tits, great tits, and coal tits) and correlated these with published, UK‐wide sparrowhawk abundances and publicly available temperature data, to determine the possible mechanisms responsible for these changes in body mass. We also examined long‐term variations in the wing lengths of these birds to test the hypothesis that changes in body mass may be accompanied by compensatory changes in wing length.

The results confirm that phenotypes of bird populations can be affected by anthropogenic factors over a relatively short time period, with potentially important ecological implications in light of predicted climatic change. Furthermore, these results demonstrate the value of citizen science in generating such large datasets for analzsing long‐term population changes.

## 2 MATERIALS AND METHODS

Data were collected by the British and Irish Ringing Scheme run by the British Trust for Ornithology (BTO), which recorded 932,722 captures of blue tits (514,235), coal tits (99,737), and great tits (318,751) in Britain and Ireland between 1965 and 2017 in which both body mass and wing length were recorded. Initial inspection of the data indicated that 16 measurements were likely to be erroneous (outside the plausible range of values), and these were therefore removed from the subsequent analysis. The remaining measurements were then sorted by month and year of capture, and multiple captures of the same individual within the same month and year were combined to reduce multiple counting of individuals, leaving 452,599 blue tits, 76,743 coal tits, and 271,641 great tits.

Individual birds were assigned to one of two age classes (adult or juvenile) based on the BTO ringing code assigned at capture. Juveniles were defined as birds of age 0, and adults were defined as birds of ages greater than 0. Birds not confirmed to be of age 0 between January 1st and June 30th were omitted from the analysis because they could potentially have been either juveniles hatched in the previous calendar year, or adults.

Data processing was performed using the Matlab computing program (Mathworks, 2016). Subsequent statistical analyses were performed in R (R Core Team, 2016). Analysis was divided into four sections: (a) A preliminary analysis of within‐year changes in body mass, to ensure that all three investigated species displayed similar patterns of body mass change and were therefore likely affected by the same environmental factors, (b) Identification of the trend in long‐term change in body mass, (c) Identification of the trend in long‐term change in wing length and (d) A linear model correlating long‐term changes in body mass with changes in environmental factors.

### 2.1 Within‐year variations in body mass

Mean body mass was calculated for each species, age class, and month to show seasonal trends in absolute body mass. A *t* test was performed for each species to determine the difference in mean annual body mass between adults and juveniles. Additionally, birds with ages coded as 3 or 5 under the BTO ringing scheme were separated from each other during this analysis to demonstrate changes in juvenile mass from fledging to adulthood. Great tits show pronounced sexual dimorphism and were therefore also separated by sex (134,442 female records and 149,666 male records, with 34,643 unsexed or inconsistently sexed records removed from the analysis) to identify differences in seasonal body mass patterns between males and females.

### 2.2 Long‐term changes in body mass

A linear regression of body mass against year was performed for each species, age class, and month to reveal any long‐term changes in body mass since the 1960s. We used these regressions to fit curves for each species and age class to describe seasonal differences in long‐term rates of change of the mean population body mass. One possibility is that apparent changes in body mass over time may be accounted for by changes in average timing of ringing over the study period, interacting with changes in bird body mass over the course of a day. To test this, a liner regression was performed for juvenile blue tits in January, including only birds caught at noon.

### 2.3 Long‐term changes in wing length

A linear regression of wing length against year was performed for each species, age class, and month to compare long‐term changes in body mass with long‐term changes in body size, using wing length as a proxy for body size (Gosler et al., 1995).

### 2.4 Linear model

A linear model was produced to identify the mechanisms responsible for the observed long‐term trends in body mass. Each body mass record was transformed into a standard deviation from the mean mass for that species and age class to allow standardized comparisons between groups. Temperatures and sparrowhawk abundances were also standardized. Based on visual similarity between the December and January datasets and among the three study species, data from all three species and both age classes in December and January were then pooled to produce a midwinter dataset, making the assumption that approximately the same pressures would be acting on each age class and species. A linear model was then fitted to determine the effects of year of capture (centered on the year 2000), mean monthly UK temperature in the year of capture (MET Office, 2018), and an index of sparrowhawk abundance in England in the year of capture (Robinson et al., 2016) on the standardized mass of the captured individuals. All variables were added both separately and as pairwise interactions, and the “dredge” function (Bartoń, 2018) was used to compare models to determine which model had the strongest Akaike information criterion (AIC) support. The three‐way interaction between all variables was not included, as it is difficult to justify biologically.

Although wing length is likely to have a major impact on individual body mass (because wing length acts as a proxy for body size, and larger birds are typically heavier; Gosler et al., 1995), wing length was omitted from this model because mean population wing length is liable to change over time in response to changing wing‐loading demands driven by changes in body mass. Consequently, inclusion of wing length in the model would be liable to mask the effects of other factors on bird body mass.

Sparrowhawk abundance indices were not available after January 2015 or before December 1974, and birds from years outside these bounds were therefore excluded from the model. Sparrowhawk abundance was recorded in the summer, and winter indices were therefore approximated by interpolating between adjacent summer measurements.

## 3 RESULTS

### 3.1 Within‐year variations in body mass

**Figure 1:**
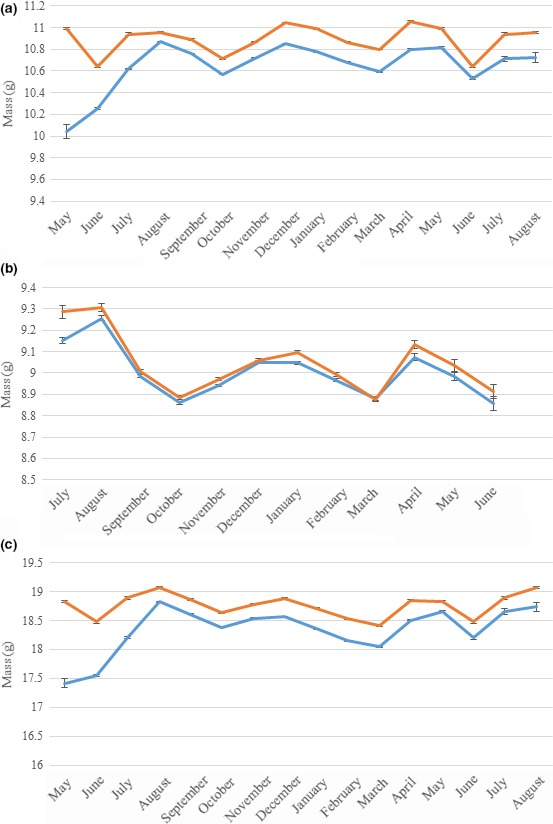
Seasonal variations in mean population body masses for adult (orange lines) and juvenile (blue lines) tits (monthly means and standard error bars). (a) Blue tits; (b) coal tits; (c) great tits. More than 12 months are included for (a) and (c) because the juvenile age class begins with individuals recorded as age 3 and then comprises individuals recorded as age 5 after December. There were fewer records for coal tits than for the other investigated species, and there were thus insufficient data for juveniles in May and June to allow those months to be plotted. Standard error bars are shown but are extremely small due to the large sample sizes

**Figure 2:**
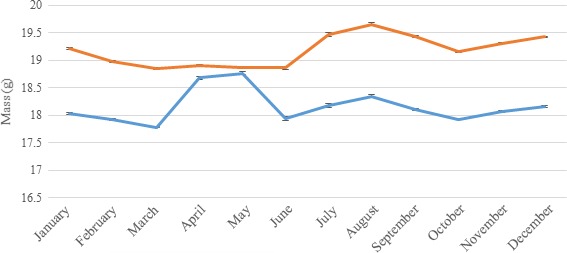
Seasonal variation in mean (and standard error) population body masses of male (orange line) and female (blue line) adult great tits. Females exhibited a clear peak in body mass in April and May that was not found in males

**Figure 3:**
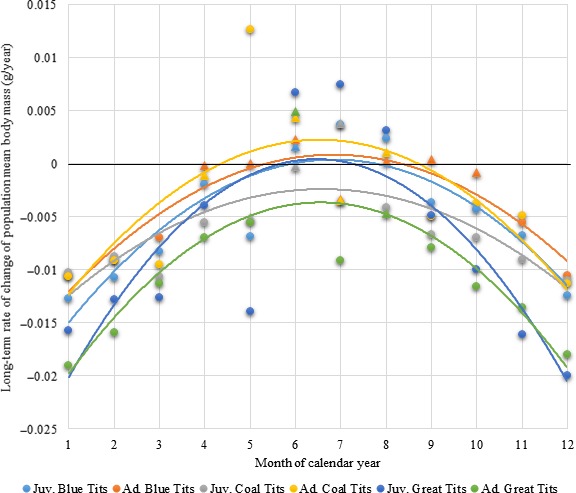
Linear regression coefficients of body mass in relation to year for all months, species and age classes. Coefficients that are significantly different from zero are presented as circles; coefficients that are not significantly different from zero are presented as triangles. Parabolic curves are fitted to each species–age class combination. “Long‐term rate of change” refers to the rate of change between the same month in one year and the next and does not reflect within‐year rate of change, as might be observed from one month to the next

**Figure 4:**
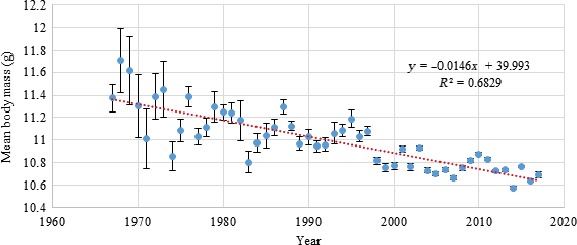
Linear regression of mean population body mass against year of capture for juvenile blue tits captured between 1967 and early 2017. The regression coefficient (−0.0146) was significantly different from 0 (*t *= −10.27, *p* < 0.001).

**Figure 5:**
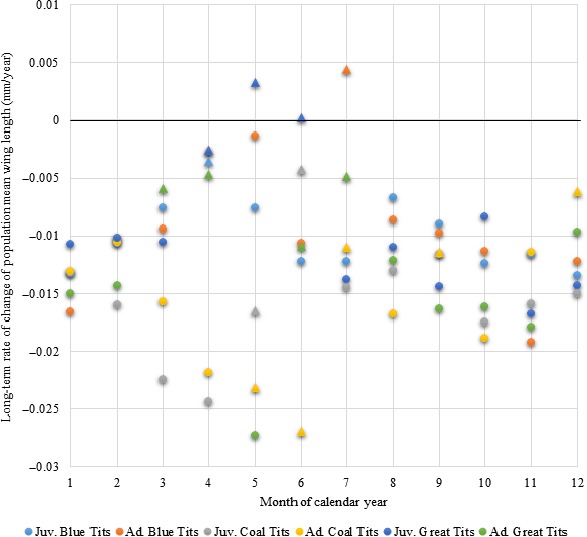
Linear regression coefficients of wing length in relation to year for all months, species, and age classes. Coefficients are significantly lower than zero for all species and both age classes in at least 7 months of the year and are not significantly higher than zero for any species or age class in any month. Coefficients significantly different from zero are presented as circles; coefficients not significantly different from zero are presented as triangles. “Long‐term rate of change” refers to the rate of change between the same month in one year and the next and does not reflect within‐year rate of change, as might be observed from one month to the next

All three species showed similar seasonal trends in body mass. Newly fledged juveniles were lighter than adults and, although this difference decreased rapidly until August, it persisted throughout the year in all species: mean 0.21 g (1.92% of mean adult body mass) in blue tits, 0.03 g (0.33% of mean adult body mass) in coal tits, and 0.35 g (1.87% of mean adult body mass) in great tits. All of these values were significantly different from zero (*t* tests, blue tits: *t *= −93.86, *df* = 300,420; coal tits: *t *= −8.77, *df* = 60,796; great tits: *t *= −76.48, *df* = 209,730; *p* < 2.2 × 10^−16^ for all species). After August, both age classes showed similar seasonal trends in body mass, with mass oscillating predictably with three separate peaks in April/May, August, and December in all species (Figures 1, 2, 3, 4, 5a–c).

When great tits were separated by sex, the April/May peak only occurred in females, while the August and December peaks occurred in both sexes (Figure 2).

### 3.2 Long‐term changes in body mass

The linear regressions of individual body mass against year (see Figure S1 in Supporting information for an example) revealed that body mass decreased in both age classes and all investigated species during a substantial proportion of the year, centered on winter (significant year‐to‐year population body mass decreases (*p* < 0.05) in all species and age classes in the months between November and March inclusive) (Figure 3). The highest rates of decrease occurred in December and January (Table 1).

**Table 1:** Mean apparent decreases in body mass in the period 1965 to 2017 for all species and both age classes during January and December expressed in absolute terms (with standard errors) and in terms of the mean body mass for the species and age class during those months, not controlling for time of capture

Species	Age class	Mean decrease in mean individual body mass ± *SE* (g/year)	Mean individual body mass (g)	Mean decrease in individual body mass (% av. body mass/year) in December & January
Blue tit	Juvenile	0.013 ± <0.001	10.82	0.1183
Coal tit	Juvenile	0.011 ± <0.001	9.05	0.1171
Great tit	Juvenile	0.019 ± <0.001	18.46	0.1007
Blue tit	Adult	0.011 ± <0.001	11.02	0.0980
Coal tit	Adult	0.011 ± <0.001	9.08	0.1179
Great tit	Adult	0.019 ± 0.001	18.80	0.1026

The linear regression of individual body mass against year, controlling for time of capture, produced a regression coefficient of −0.009561 g/year. This is slightly smaller than the coefficient produced without this control (−0.01275 g/year), but is still highly significantly different from zero (*p* = 9.35 × 10^−11^).

Because numbers of captures have increased in recent years, the linear regressions used to calculate long‐term rates of change in mean body mass could be disproportionately affected by trends in recent years. However, analysis of long‐term rates of change of mean body mass using only the mean masses in each January rather than all the individual captures during January suggests that the decline was not a purely recent event. The relevant graph for juvenile blue tits is shown in Figure 4.

### 3.3 Long‐term changes in wing length

Linear regressions revealed that mean wing length decreased in both age classes and all investigated species (Figure 5). Both age classes in all three species showed significant decreases (*p* < 0.05) in mean population wing lengths in at least 7 of the 12 months of the year, and neither age class in any species showed a significant increase in mean wing length in any month of the year. These rates of decrease in mean wing length showed far less seasonality than the corresponding rates of decrease in mean population body mass.

### 3.4 Linear model

Model selection using the dredge function identified strongest support for the model that contained all of the variables and interaction terms (Table 2; AIC weight = 0.995, AIC_c_ difference between most supported and second‐most supported model = 10.7). The model selection table is Table S1 in Supporting information. Individual variation in masses and wing lengths was sufficiently high that the most supported model has very low explanatory power on an individual scale (*R* ^2^ = 0.02106). However, a regression of average masses in each year against time (Figure 4, *R* ^2^ = 0.6829) shows that explanatory power is likely to be much higher on a population scale.

**Table 2:** Linear model terms for the single model most highly supported by AIC

Variable	Coefficient	*t* value	*p*
Intercept	0.2330 ± 0.0034	64.761	<2 × 10^−16^
Year	−0.0147 ± 0.0004	−37.855	<2 × 10^−16^
Temperature	−0.0897 ± 0.0039	−22.883	<2 × 10^−16^
Sparrowhawk abundance	−0.0038 ± 0.0015	−2.647	8.12 × 10^−3^
Year: temperature	0.0011 ± 0.0003	3.564	3.65 × 10^−4^
Year: sparrowhawk abundance	0.0007 ± <0.0001	7.265	3.74 × 10^−13^
Temperature: sparrowhawk abundance	−0.0172 ± 0.0016	−10.551	<2 × 10^−16^

*R* ^2^ = 0.02106, *df* = 197,010.

## 4 DISCUSSION

Our study demonstrates that multiple garden bird species have undergone a slow but steady decrease in body mass and wing length over the past 60 years, which is associated with increasing winter temperatures and predator abundance.

In terms of within‐year variation, all three investigated species displayed similar patterns of annual body mass change, suggesting that they were all likely to be affected by the same factors. Furthermore, it seems likely that the sexual dimorphism in annual body mass change apparent in great tits would apply in all three species, given that the April/May peak in female body mass can be attributed to egg production (Perrins, 1979).

Regarding longer‐term changes, body mass decreased only in the winter months in all three species and both age classes. Some of this change may be accounted for by change in the mean capture times of birds. However, much of it is not. There are several possible explanations for this unexplained change. Temperatures are currently increasing across Britain due to global warming (Jenkins et al., 2008), and the energy‐storage benefit of body fat to birds during the winter may thus be decreasing, leading to a lower optimal winter body mass in response to constant predation risk. The current analysis showed that winter temperature had a strong effect on body mass in all three species, with birds being lighter in warmer winters, which supports this hypothesis.

Optimal body mass could also be reduced by increased predation pressure (Gosler et al., 1995), even in the absence of changes in temperature. However, the sparrowhawk is one of the main predators of all three tit species (Perrins, 1979), and sparrowhawk abundance in Britain plateaued in the 1990s (Robinson et al., 2016), when the population recovered from the negative effects of the pesticide DDT after its use was banned in Britain in 1984 (Newton, 1986). Furthermore, increasing predator abundance cannot explain why prey body mass would only decrease in winter, given that although passerine food availability is likely to vary with season, their predators must hunt at all times of the year.

Finally, increasing levels of human supplementary feeding of garden bird species across Britain (Plummer et al., 2015) may also reduce the energy‐storage benefit of body fat to birds during the winter by increasing the reliability of food availability, which may also lead to a reduction in optimal body mass in response to constant predation risk. We also demonstrated a relationship between winter body mass and calendar year that was independent of the effect of winter temperature or sparrowhawk abundance, suggesting that a long‐term increase in supplementary feeding of garden birds (Plummer et al., 2015) may also have influenced the trade‐off between starvation risk and predation risk.

Previous studies showed that great tits have undergone an evolutionary change in bill morphology in response to human supplementary feeding (Bosse et al., 2017), and that populations are often locally adapted to climate, even on quite small scales (Morrison, Robinson, & Pearce‐Higgins, 2016). It is now well known that human activities impose sufficient selection pressures on some wild species to allow evolution on directly observable timescales (Allendorf, England, Luikart, Ritchie, & Ryman, 2008). Short‐timescale evolution could thus be responsible for the observed changes in other aspects of tit morphology. However, it is also possible that the changes observed here are attributable to individuals’ plastic regulation of their body mass in response to changing environmental conditions (Gosler et al., 1995).

Wing length is under genetic control (Lessells & Ovenden, 1989), and long‐term changes in mean population wing lengths could thus be the result of evolution. For any individual bird, wing length changes over the course of a year are predictable and are caused by a combination of feather wear at the wingtips (Vágási, Pap, Tökölyi, Székely, & Barta, 2011) and molting. Although wing length is unlikely to be influenced by daily or even monthly plastic regulation, it could theoretically be influenced by yearly plastic regulation if the physical properties of the feathers change adaptively during molting to optimize them for the environment in which the bird “predicts”; it is likely to exist in the coming year. However, there is currently no evidence for such a process and therefore we suggest that the observed reduction in wing length in all three species is the result of short‐timescale evolution.

The observed reduction in wing length in all the investigated species is likely to represent a response to the reduced optimal body mass, given that individuals with larger mass require longer wings to maintain their wing loading (Andrews, Mackenzie, & Gregory, 2009). Although the reduction in body mass was only evident in the winter, individuals cannot manipulate their wing length physiologically to the same degree as their body mass, and wing length should thus be optimized to the mean body mass throughout the year, or to a weighted mean if selection on wing length is more intense at certain times of year than at others. Mean population wing length may thus be expected to decrease significantly from year to year in response to changes in body mass, as shown by our data, even in months when the mean population body mass does not decrease.

The linear model used to determine the factors influencing body mass was somewhat crude, in that the temperature and sparrowhawk abundance predictor variables were averaged over large areas and may therefore not always be correct for the individual birds in the sample (which will be affected by local temperatures and predator abundances). However, the use of sparrowhawk abundance data from England was justified given that most of the birds (812,252 of 933,024 captures) in the analysis were recorded in England.

The results of the model supported existing theories very well: higher temperatures and higher sparrowhawk abundances decreased the mean body masses of the birds, presumably by reducing the benefits of fat as an energy‐storage medium or by increasing the cost of fat in terms of predation risk, respectively (McNamara & Houston, 1990).

It is possible that the significant decrease in bird body mass over time independent of temperature or sparrowhawk abundance may be an artifact resulting from the coarse scales of the measurements of temperature and sparrowhawk abundance. However, it is also possible that the decrease may be at least partly due to other variables, such as the level of supplementary feeding, which has been increasing in Britain and Ireland (Plummer et al., 2015) and which provides a reliable winter food source for many passerine species, reducing the benefits of additional body fat for energy storage and therefore reducing their optimal winter body mass.

The interaction terms including an effect of year indicated a decline in the magnitudes of the effects of both temperature and sparrowhawk abundance over time. The interaction between sparrowhawk abundance and temperature indicated that an increase in one increased the magnitude of the effect of the other. This also supports existing theories: fat gain represents a trade‐off between predation risk and energy storage (Krams et al., 2010; McNamara & Houston, 1990; Newton, 1969, 1998). Consequently, when the costs of fat gain (in terms of increased predation risk) are very low (e.g., because sparrowhawk abundance is low), there is less pressure to maintain a low body mass when possible (e.g., at higher temperatures where energy reserves are less important), and body mass would thus be predicted to respond less to temperature when sparrowhawk abundance is low, as observed.

This study was limited by the coarse scale of measurement of data. Consequently, our findings warrant further detailed research to examine the effects of climate change at smaller scales, and to clarify the effects of other potential factors that could affect optimal body mass in birds, such as levels of supplementary feeding. Some proportion of the observed change in bird body mass over time also appears to be accounted for by a change in the timing of bird capture, with captures becoming, on average, earlier in the day in more recent years. This can be regarded as an artifact. Further studies are also needed to determine to what degree the observed pattern is reflected in other countries and ringing schemes, and in bird species less dependent on garden feeders in winter.

Large, pre‐existing datasets are an underutilized resource in ecology, and with some exceptions (McLean, Jeugd, & Pol, 2018) are rarely exploited to their full potential. The current study demonstrates the ability of data, gathered with no specific hypotheses in mind, to test specific hypotheses. Our results using such a dataset provide the first evidence for significant declines in the winter body masses of three common garden bird species throughout Britain and Ireland over the last 50 years, with annual variations correlated with climate warming. Such trends are thus liable to persist if winters continue to become warmer due to climate change, and studies in other taxa have demonstrated the potential for changes in population mean body mass to influence ecosystem functioning (Audzijonyte, Kuparinen, & Fulton, 2014) and life histories (Kuparinen & Hutchings, 2012). Consequently, our results represent an important point of consideration for conservation and future ecological studies.

## CONFLICT OF INTEREST

The authors declare that there are no conflicts of interest.

## AUTHOR CONTRIBUTIONS

EF conceived this study, analyzed the data, and prepared the manuscript for submission. RR contributed to the analysis and interpretation of the results. Both authors contributed critically to the drafts and gave final approval for publication.

## Data Availability

Data used in these analyses are archived in Dryad: https://doi.org/10.5061/dryad.1d21m78. Up to date data are available from the BTO upon request.
