# Supplementary figures and images for: Long‐term declines in winter body mass of tits throughout Britain and Ireland correlate with climate change
Source: Ecol Evol. 2018 Dec 26;9(3):1202–10. doi: 10.1002/ece3.4812 (PMC6374658; doi:10.1002/ece3.4812)

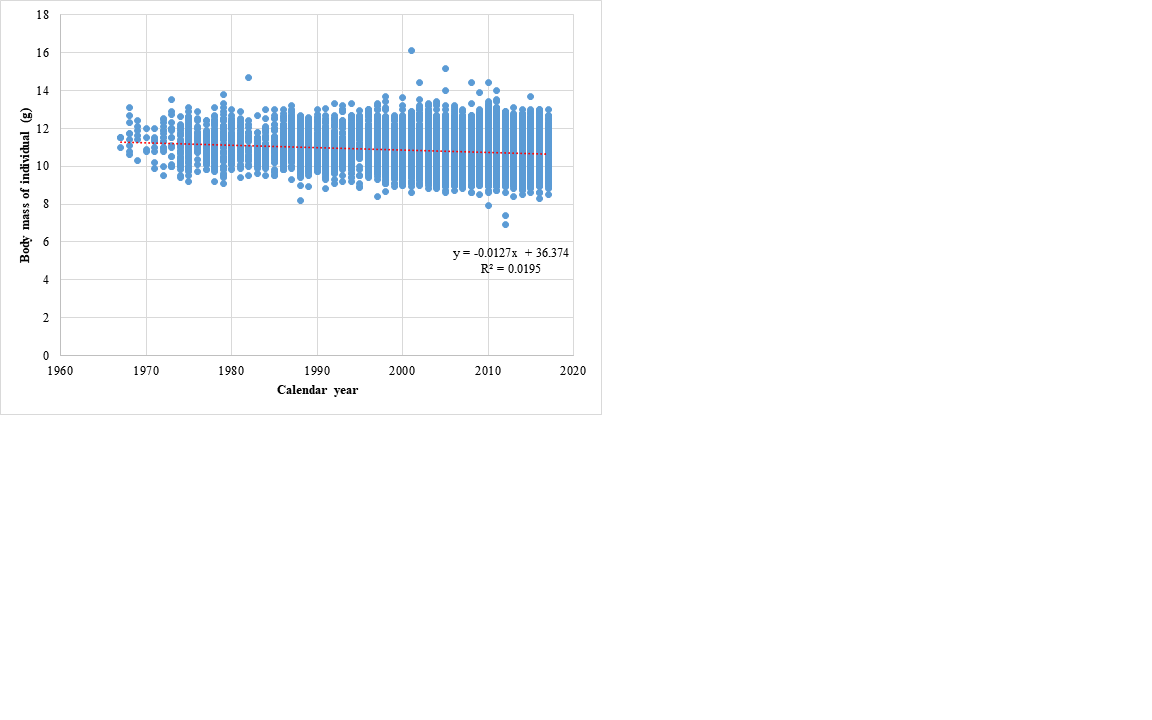

Supplement: Supplementary file 1 [file ECE3-9-1202-s001.tif]
