# Supplementary material for: Long‐term declines in winter body mass of tits throughout Britain and Ireland correlate with climate change
Source: Ecol Evol. 2018 Dec 26;9(3):1202–10. doi: 10.1002/ece3.4812 (PMC6374658; doi:10.1002/ece3.4812)
Supplement: Supplementary file 2 [file ECE3-9-1202-s002.docx]

| **Predictor in model?** | | | | | |  |  |
| --- | --- | --- | --- | --- | --- | --- | --- |
| Year | Spar. | Temp. | Year*Spar. | Year*Temp. | Spar.*Temp | **df** | **AICc** |
| X | X | X | X | X | X | 8 | 551887.3 |
| X | X | X | X |  | X | 7 | 551898.0 |
| X | X | X |  | X | X | 7 | 551938.1 |
| X | X | X |  |  | X | 6 | 551946.5 |
| X | X | X | X | X |  | 7 | 551996.6 |
| X | X | X | X |  |  | 6 | 551999.1 |
| X | X | X |  | X |  | 6 | 552018.6 |
| X | X | X |  |  |  | 5 | 552020.7 |
| X |  | X |  | X |  | 5 | 552038.2 |
| X |  | X |  |  |  | 4 | 552044.9 |
| X | X |  | X |  |  | 5 | 552919.2 |
| X | X |  |  |  |  | 4 | 552959.4 |
| X |  |  |  |  |  | 3 | 552960.5 |
|  | X | X |  |  | X | 5 | 554683.5 |
|  | X | X |  |  |  | 4 | 554684.0 |
|  |  | X |  |  |  | 3 | 554743.6 |
|  | X |  |  |  |  | 3 | 555914.5 |
|  |  |  |  |  |  | 2 | 556074.0 |
